# Experimental Comparison between Ethanol and Hexane as Solvents for Oil Extraction from Peanut Press Cake

**DOI:** 10.3390/foods12152886

**Published:** 2023-07-29

**Authors:** Paloma Jamily Cristina Magalhães, Daniel Gonçalves, Keila Kazue Aracava, Christianne Elisabete da Costa Rodrigues

**Affiliations:** 1 Laboratory of Separation Engineering (LES), Department of Food Engineering (ZEA), School of Animal Science and Food Engineering (FZEA), University of São Paulo (USP), P.O. Box 23, Pirassununga 13635-900, Brazil; palomajcmagalhaes@usp.br (P.J.C.M.); k.aracava@usp.br (K.K.A.); 2 Food Technology Laboratory (LTA), Center for Agricultural Sciences and Technologies (CCTA), State University of Northern Rio de Janeiro (UENF), Campos dos Goytacazes, Rio de Janeiro 28013-602, Brazil; danielg@uenf.br

**Keywords:** *Arachis hypogaea* L., solid-liquid extraction, renewable solvent, physical properties, density, viscosity, nitrogen solubility index, fatty acid composition, triacylglycerol composition

## Abstract

Ethanol (Et) has been suggested as a substitute for hexane (Hx) for use in the extraction of oils from different oleaginous matrices. In this study, Et and Hx were used to extract the residual oil present in a peanut press cake (PPC). Certain variables, such as temperature, solid/solvent ratio and the number of contact stages, in the sequential cross-current extraction process were evaluated; additionally, the effects of these variables on oils (POEt and POHx) and defatted solids (DSEt and DSHx) were explored. Hx exhibited an extraction yield of 86 ± 2% in two stages at 55 °C and a solid/solvent mass ratio of 1/4. Compared with Hx extraction, to achieve an Et extraction yield of 87 ± 4%, it was necessary to use a higher temperature (75 °C), a greater amount of solvent (solid/solvent ratio of 1/5) and a greater number of contact stages (3). POEt and POHx presented compositions in terms of fatty acids and triacylglycerols and physical properties similar to that of cold-pressed peanut oil (CPPO). POEt showed a more intense green/yellow hue and higher free acidity (1.47 ± 0.03%) than POHx and CPPO (0.82 ± 0.04 and 0.43 ± 0.02 free acidity mass %, respectively), indicating that the deacidification and bleaching steps in refining should be encumbered. DSEt and DSHx exhibited high protein contents (>45% by mass) and nitrogen solubilities (86 ± 6 and 98 ± 1%, respectively), indicating that they could be used to obtain proteins.

## 1. Introduction

Peanut (*Arachis hypogaea* L.) is produced worldwide, and it is widely consumed in many forms, including roasted grain, peanut butter, and peanut oil [1]. The world production yields of peanut grain, oil and meal in May 2023 were 50.4, 6.5 and 7.9 thousand metric tons (KMT), respectively. The four main world producers are China, India, Nigeria, and the USA, which are distributed in Asia, Africa, and America. These countries are responsible for 63.5% of the world production of peanut grain, 75.3% of peanut oil and 75.8% of peanut meal/cake [2].

The peanut has a similar composition to the walnut, and is considered an oilseed due to its high lipid content (40–50% by weight). The oil has a balanced composition in terms of fatty acids since it is primarily composed of monounsaturated and polyunsaturated fatty acids. The lipid profile associated with bioactive compounds, such as resveratrol and phytosterols, may provide benefits to human health, such as anti-inflammatory activity and risk reduction for cancer and cardiovascular disease [1].

New genotypes have been developed with advantageous traits, including oils with relatively low iodine values (IVs) between 86 and 107 g iodine/100 g oil due to their relatively high oleic acid contents [3,4]. The percentage of this monounsaturated fatty acid in regular grains can vary between 41 and 67%, while in grains classified as high-oleic, it can reach 80% [4]. Oil with a high oleic acid content and low IV has a long shelf life due to its great oxidative stability [5].

Due to its high lipid content, peanut oil is traditionally obtained by mechanical pressing, and the process can be associated with solvent extraction to recover the residual oil contained in the press cake [6]. The solvent industrially used for the extraction of vegetable oils, known as hexane, is derived from the distillation of naphtha, which is a relatively light fraction of petroleum that is composed of a mixture of n-hexane isomers.

Although this solvent has various advantages, such as a high oil dissolution capacity and easy recovery for reuse in the process, its use may be associated with environmental and human health risks. N-Hexane can be classified as a volatile organic compound (VOC) due to its high vapor pressure and low water solubility. The high volatility of n-hexane can lead to losses of this component to the atmosphere during the industrial processing of oilseeds, contributing to environmental pollution [7]. In addition, the major isomer n-hexane and its metabolites have been associated with chronic toxicity (neurotoxicity and reprotoxicity). N-Hexane affects workers due to occupational exposure, and the general population via its ingestion as a residue in food [8].

Several substances have been evaluated as alternative solvents to hexane, including ethanol. This solvent is noteworthy due to the low toxicity relative to hexane and the possibility of being obtained from renewable sources [9]. According to the *Food and Drug Administration* (FDA) [10], ethanol is classified as Class 3 in terms of toxicity, i.e., less toxic than hexane, which is classified as Class 2. In the USA, which is the world’s largest producer, ethanol is obtained from corn; in Brazil, which is the second largest producer in the world, ethanol is mainly produced from sugarcane. Together, the USA and Brazil were responsible for 82% of the 27.3 billion gallons of ethanol produced in the world in 2021 [11].

Ethanol has been proposed for the extraction of lipids from oilseeds directly subjected to solvent extraction due to their moderate lipid content, such as soybean [12] and rice bran [13], and for the extraction of oil remaining in the cakes of Brazil nuts [14], babassu [15], baru kernels [16], sunflower seeds [17], and macadamia nuts [18]. Press cakes of sunflower and pumpkin seeds have been subjected to the extraction of residual oil using ethanol and hexane as solvents [19]. The authors have observed a relatively great amount of residual oil in the solids obtained from extraction with ethanol and a great extraction of pigments with the use of the renewable solvent. Studies on the extraction of residual oil from macadamia nut [18] and baru almond [16] press cakes show that the oil extraction yield is dependent on the number of contact stages and the temperature in the sequential extraction. The authors have evaluated the effect of the solvent on the characteristics of the defatted solid, and the solubility of nitrogen present in the protein fraction decreases with increasing temperature in the oil extraction step.

Regarding peanuts, Arnold and Choudhury [20] and Desmarina et al. [21] have compared the performance attributes of hexane and ethanol solvents for the extraction of the oil present in grains without prior mechanical pressing. In general, both solvents are effective in oil extraction [21,22], and ethanol is responsible for extracting a great amount of nonlipid compounds [20].

Studies evaluating the extraction of residual oil from peanut press cake are scarce. Fonseca and Regitano-d’Arce [23] have evaluated the ability of ethanol to extract aflatoxins from peanut cake concomitantly with oil extraction at different hydration degrees (0, 4, 7 and 10% water by volume). The authors have observed that hydrated solvents are effective for the removal of aflatoxins; however, the higher the water content in ethanol is, the lower the lipid extraction capacity. Tate et al. [24] evaluated the performance of hexane in the extraction of residual oil in peanut cake with the subsequent use of hydroalcoholic solution (80% ethanol) to remove pigments and the bitter taste. The authors have evaluated the application of solid defatted with hexane as a partial replacement of wheat flour for the formulation of cookies, showing that the byproduct of oil extraction can be used in food preparation for increasing the protein content. According to Pattee [5] and Arya et al. [1], the residual solid obtained from the extraction of peanut oil with a solvent, notably hexane, is used as animal feed to prepare soups and bakery products, such as cookies, as proposed by Tate et al. [24].

Based on the above information, it is necessary to compare hexane and ethanol for the extraction of residual oil from peanut press cake. Parameters related to the extraction process, such as the temperature, solid/solvent ratio and configuration of the extraction in terms of the number of contact stages, are necessary to support decision-making regarding the replacement of the fossil solvent by a renewable solvent. In addition, the products of extraction using solvent, oil and defatted solid must be characterized and compared to predict subsequent oil refining operations and possible applications for the defatted solid.

In this context, this study aimed to evaluate the conditions of ethanol use in the extraction of residual oil from peanut cake. For this objective, the physicochemical parameters of the oil obtained with ethanol were compared with those of the oils from mechanical pressing and extraction with hexane. The oils were extensively characterized in terms of fatty acid and triacylglycerol composition, free acidity, density, viscosity, surface tension and color. The solids defatted by both solvents were evaluated to determine the impacts of ethanol on the protein content and nitrogen solubility index of the protein fraction.

## 2. Materials and Methods

### 2.1. Materials

The cake and oil from the cold pressing of raw peanuts were purchased from Sr. Ouro Verde (Almirante Tamandaré, Paraná, Brazil). The reagents used in the experiments and analyses were n-hexane (CAS 110-54-3), absolute ethanol (CAS 64-17-5), glacial acetic acid (CAS 64-19-7), anhydrous sodium carbonate (CAS 497-19-8), hydrochloric acid (CAS 7647-01-0) and sodium hydroxide (CAS 1310-73-2), which were purchased from Synth (Diadema, Sao Paulo, Brazil). Standards of glucose (CAS 50-99-7), gallic acid (CAS 149-91-7), (+)-catechin (CAS 154-23-4), methyl tridecanoate (CAS 1731-88-0), 4-(dimethylamino)cinnamaldehyde (DMAC—CAS 6203-18-5) and 2M Folin–Ciocalteu solution were purchased from Sigma-Aldrich (St. Louis, MO, USA). Acetone (CAS 67-64-1) was purchased from Êxodo Científica (Sumaré, Sao Paulo, Brazil). All reagents had a purity ≥ 98.0%, as reported by the suppliers.

### 2.2. Methods

#### 2.2.1. Peanut Press Cake Characterization

Peanut press cake (PPC) was characterized in terms of particle size distribution using a sieve system (Tyler series, Wheeling, WV, USA), and it was possible to calculate the mean particle diameter according to the ASAE method [25].

The bed porosity (ε) of PPC was calculated using Equation (1), where d_A_ is the apparent density, as determined by measuring the mass (g) of PPC occupying a certain volume (mL) using previously calibrated glassware, and d_T_ is the true density obtained by pycnometry with helium gas (Quantachrome Instruments, MVP-6DC, Boynton Beach, FL, USA).

(1) $$\varepsilon \left(\%\right)=\left(1-\frac{d_{A}}{d_{T}}\right)\times 100$$

PPC was analyzed for moisture (method Ac 2-41, [26]) in a forced convection oven (Nova Orgânica, N035/3, Piracicaba, Sao Paulo, Brazil) at 130 °C for 4 h. The lipid content (method Am 5-04 [26]) of the dried material was determined in a semiautomatic extraction system (Ankom, XT10, Macedon, NY, USA) at 90 °C for 5 h using n-hexane as the solvent. The total nitrogen content (method Ba 4e-93 [26]) was analyzed in a combustion nitrogen analyzer (Leco, FP-528, St. Joseph, MI, USA) using a factor of 5.46 [27] to convert the total nitrogen content into proteins.

The ash (900.02 [28]) was quantified by muffle incineration (Marconi, MA385/3, Piracicaba, Sao Paulo, Brazil). Soluble and insoluble fibers were determined by the enzymatic method [29], and the nonfibrous carbohydrate content was estimated by the difference. The presence of mycotoxins (B1, B2, G1, G2 and ochratoxin) was investigated by liquid chromatography (LC)-mass spectrometry (MS) following the method proposed by Sulyok et al. [30] with modification by Franco et al. [31].

PPC was characterized in terms of total phenolic compounds (TPC) and total flavanols (FLA). The extraction of TPC and FLA content was performed according to the method described by Ortega et al. [32]. The compounds were extracted with a solution of acetone, water and glacial acetic acid (70/29.5/0.5, *v*/*v*/*v*) by adding 5 mL of solution to 0.5 g of solid sample previously cold-defatted with hexane (PPC/hexane ratio of 1/3 at 3 contact stages).

The dispersions of defatted PPC in acidified acetone solution were stirred for 5 min, followed by an ultrasonic bath (Unique, UltraCleaner 1400, Indaiatuba, Sao Paulo, Brazil) at 37 °C for 10 min. A sequence of shaking for 5 min and resting for 25 min was repeated three times. The samples were centrifuged (Thermo, CR3i, Waltham, MA, USA) at 3000× *g* for 5 min at 25 °C. The supernatant was collected and filtered with an Allcrom nylon filter syringe with a 0.45 µm opening.

TPC was determined by the Folin–Ciocalteu method described by Singleton, Orthofer and Lamuela-Raventos [33], adopting the calibration curve for gallic acid, where the results are expressed in mg gallic acid equivalent/g PPC (mg GAE/g PPC). Thus, 0.5 mL of the extracts diluted in water were added to 2.5 mL of 10% Folin–Ciocalteu solution and 2.0 mL of 7.5% sodium carbonate solution. After 2 h in the dark, the absorbance was measured in a spectrophotometer (Shimadzu, UV-1650 PC, Kyoto, Japan) at 760 nm.

The 4-(dimethylamino)cinnamaldehyde (DMAC) method was described by Okiyama et al. [34]. For the calibration curve, the standard (+) catechin was used, where the results are expressed as mg catechin equivalent/g PPC (mg CE/g PPC). Thus, the reaction was performed directly in the reading cuvette by adding 0.5 mL of the extracts diluted in ethanol and 2.5 mL of DMAC reagent. The absorbance was measured over the reaction time in a spectrophotometer (Shimadzu, UV-1650 PC, Japan) at 640 nm until the maximum absorbance value was obtained.

#### 2.2.2. Cold-Pressed Peanut Oil Characterization

The cold-pressed peanut oil (CPPO) was characterized in terms of fatty acid composition by the fatty acid methyl ester (FAME) derivatization method (Ce 2-66 and Ce 1-62 [27]). The analysis was performed in a gas chromatograph (Shimadzu GC-2010, Japan) with an automatic injector (Shimadzu AOC 20i, Japan) and flame ionization detector, as described by Sawada et al. [35]. Fatty acids were identified by comparison with external standards (Supelco, Merck KGaA, Darmstadt, Germany). Quantification was based on the comparison of the area ratios of each fatty acid with the area of the internal standard (methyl tridecanoate, C13:0). Correction factors for the response of the flame ionization detector and the conversion of fatty acid methyl esters to fatty acids were used [35].

The probable composition of triacylglycerols (TAGs) was estimated from the fatty acid profile using the statistical procedure suggested by Antoniosi Filho et al. [36], as executed in MATLAB software (MathWorks Inc., Natick, MA, USA).

The free acidity of the oil was determined by titration (2201 [37]) with a 0.01 M NaOH alkaline solution in an automatic burette (Metrohm Dosimat 775, Herisau, Switzerland). Moisture was quantified by Karl Fischer titration (method Ca 2e-84 [26]) using a KF Titrino titrator (Metrohm 787 KF Titrino, Herisau, Switzerland). The color of CPPO was characterized in a noncontact reflectance spectrophotometer (HunterLab, Eros, Blacksburg, VA, USA). The hue angle (°h) (Equation (2)) and the total color change (ΔE) (Equation (3)) were calculated according to the methodologies suggested by McGuire [38] and Li et al. [39], respectively. The values determined for CPPO were used as a reference to calculate the ΔE for peanut oils obtained via solvent extraction (Section 2.2.3).

(2) $${}^{\circ}h=\tan^{-1}\frac{b^{*}}{a^{*}}$$

(3) $$\Delta E=\sqrt{{\left(L^{*}-{\;L}_{\mathrm{CPPO}}^{*}\right)}^{2}+{\left(b^{*}-{\;b}_{\mathrm{CPPO}}^{*}\right)}^{2}+{\left(a^{*}-{\;a}_{\mathrm{CPPO}}^{*}\right)}^{2}}$$

where L* is the luminosity, a* is the red/green coordinate and b* is the yellow/blue coordinate.

The physical properties of density, dynamic viscosity and kinematic were determined in a Stabinger viscometer (Anton Paar, SVM 3000, Graz, Austria) in the temperature range between 20 and 70 °C. The refractive index was determined at 25 °C in a portable digital refractometer (Atago, PAL-RI, Ribeirao Preto, Sao Paulo, Brazil), and the surface tension was measured in a tensiometer (Attension, Sigma 702, Helsinki, Finland) at 20, 50 and 70 °C.

#### 2.2.3. PPC Oil Extraction with Ethanol and Hexane

PPC was subjected to the extraction of the lipids remaining from pressing with the solvents absolute ethanol (Et) and n-hexane (Hx) in a stainless-steel batch extractor (500 mL) (Tecnal, TE-139-E4, Piracicaba, Sao Paulo, Brazil), as described by Oliveira et al. [40]. The extractor is sealed to avoid solvent losses, equipped with a manometer, mechanical agitator, temperature controller, basket for packing the peanut press cake, and a valve located on the bottom of the apparatus that allows the withdrawal of the extract phase. Constant agitation at 180 rpm and an extraction time of 1 h were adopted for all experiments, as described by Navarro and Rodrigues [18]. The temperature conditions and solid-to-solvent mass ratio (*w*/*w*) varied according to the type of solvent: Et or Hx.

For ethanol, preliminary experiments were performed in a single stage at temperatures of 60, 75 and 90 °C while considering a solid/solvent mass ratio of 1/5. The aim was to evaluate which temperature would provide the highest oil extraction yield with less impact on the proteins in the defatted solids (DSs); this objective was completed by determining the nitrogen solubility index (NSI) [41] according to the methodology described in Section 2.2.4.

Based on these preliminary experiments, the temperature of 75 °C was selected for the subsequent experiments of sequential extraction with Et in the cross-current configuration. The aim of the multistage experiments was to evaluate the number of solid/solvent contact stages required to achieve a given oil extraction yield. In this type of operation and configuration, after the first stage of contact between PPC and solvent, the solid partially defatted from the first stage became the feedstock for the subsequent stage, contacting the new solvent, as demonstrated by Toda et al. [42]. In the sequential extraction experiments performed with Et, a solid/solvent mass ratio of 1/5, a contact stage number of 5, a constant agitation speed of 180 rpm and an extraction time of 1 h at each stage were adopted.

Regarding the solvent Hx, the extraction experiments were performed at 55 °C, which is the temperature commonly used for the industrial extraction of vegetable oils for this solvent [43]. In this case, in the sequential extraction in cross currents, 2 contact stages of 1 h duration, 180 rpm constant agitation and 1/4 solid/solvent mass ratio were evaluated.

For all extraction experiments, the system components—PPC, or DS and solvent—were weighed on a precision analytical balance (0.0001 g, Adam, PW-254, Milton Keynes, U.K.) respecting the pre-established solid/solvent mass ratio.

The extraction experiments were performed at least in duplicate. Two phases were obtained from each extraction stage, the liquid phase (extract) and solid phase (DS), which were subjected to the characterizations described in Section 2.2.4.

#### 2.2.4. Characterization of Extract and Defatted Solid Phases

After each extraction step, the solid phase (DS) was weighed on a semi analytical balance (0.01 g, Adam, PGW-1502i, Milton Keynes, U.K.) to determine the amount of solution adhered (static liquid holdup or retention index (RI) expressed as kg of adhered solution/kg of inert solid).

Samples of the DS obtained at each stage, for both Et and Hx, were desolventized in a forced convection oven (Nova Orgânica, N035/3, Piracicaba, Sao Paulo, Brazil) at 60 °C for 24 h, and then in a vacuum oven (Tecnal, TE395, Piracicaba, Sao Paulo, Brazil) at 50 °C for 4 h and 21 kPa. The residual oil (RO) and protein contents in the DSs were determined according to the official methods (Am 5-04 and Ba 4e-93, respectively [26]) described in Section 2.2.1. Based on the lipid contents in the CPPO and DS, it was possible to calculate the oil extraction yield (*Y*, in %) (Equation (4)):

(4) $$Y\;\left(\%\right)=\frac{M^{\mathrm{PPC}}\times \left(w_{\mathrm{oil}\;}^{\mathrm{PPC}}\right)-M^{\mathrm{DS}\;}\times \left(w_{\mathrm{oil}}^{\mathrm{DS}}\right)}{M^{\mathrm{PPC}}\times \left(w_{\mathrm{oil}\;}^{\mathrm{PPC}}\right)}\times 100$$

where M^PPC^ corresponds to the mass of raw material, $w_{\mathrm{oil}\;}^{\mathrm{PPC}}$ corresponds to the mass fraction of oil in the raw material, M^DS^ corresponds to the defatted solid mass and $w_{\mathrm{oil}}^{\mathrm{DS}}$ corresponds to the mass fraction of residual oil present in the degreased solid from each stage.

The protein solubility indices of the desolventized DSs, expressed as the NSI, were determined at 25 °C by using water as a solvent at pH 9.0, according to the methodology of Morr et al. [41] (Equation (5)):

(5) $$NSI\;\left(\%\right)=100\times \frac{N^{\mathrm{liquid}\;\mathrm{phase}}\times {\;M}^{\mathrm{water}}}{{\;N}^{\mathrm{sample}}\times {\;M}^{\mathrm{sample}}}$$

where $N^{\mathrm{liquid}\;\mathrm{phase}}$ corresponds to the nitrogen content in the filtered sample (%); $N^{\mathrm{sample}}$ corresponds to the nitrogen content in the solid sample (%); $M^{\mathrm{sample}}$ corresponds to the sample mass (g); and $M^{\mathrm{water}}$ corresponds to the mass of water (g).

The water contents of the extracts from each of the extraction stages were determined by Karl Fischer (KF) titration (method Ca 2e-84, [26]) using a KF Titrino titrator (Metrohm 787 KF Titrino, Herisau, Switzerland).

Additional characterizations of the oils extracted with Et at 75 °C and Hx at 55 °C were performed; these oils were named POEt and POHx, respectively. Thus, for this additional characterization, the extracts obtained from the sequential stages of extraction with Et and the sequential stages with Hx were pooled and subjected to desolventization in a rotary evaporator (Heidolph Instruments, model Hei-VAP Silver, Schwabach, Germany) at 50 °C and 7 kPa, followed by drying in a vacuum oven (Tecnal, TE395, Piracicaba, Sao Paulo, Brazil) at 50 °C and 21 kPa for 24 h for POHx and 48 h for POEt. These oils were characterized in terms of fatty acid composition, triacylglycerol composition, free acidity, density, viscosity, refractive index and surface tension, as described in Section 2.2.2.

The DSs from the sequential cross-current extraction with Et and Hx, called DSEt and DSHx, respectively, were subjected to additional characterization in terms of moisture, lipids, proteins, ash, nitrogen solubility index and TPC and FLA contents, as previously described in Section 2.2.1. To enable these analyses, the residual solvent contained in the respective DSs was removed in a vacuum oven (Tecnal, TE395, Piracicaba, Sao Paulo, Brazil) at 50 °C for 4 h and 21 kPa.

#### 2.2.5. Statistical Analysis

The extraction experiments were performed at least in duplicate, while the characterizations were performed in triplicate or quintuplicate. The results were evaluated for significance (*p* ≤ 0.05) using Duncan’s multiple comparisons test with SAS^®^ software (version 9.3, SAS Institute Inc., Cary, NC, USA) and *t*-tests using R Core Team software (version 4.2.1, The R Foundation, Vienna, Austria, 2022).

## 3. Results and Discussion

### 3.1. PPC Characterization

The moisture content of the PPC was 4.4 ± 0.1% by mass. The chemical composition was 49.5 ± 0.3% lipids, 28 ± 1% protein, 2.1 ± 0.1% ash and 20 ± 1% total carbohydrates, estimated by difference. The fiber contents were 1.7 ± 0.1% soluble fiber and 3.9 ± 0.1% insoluble fiber (% by mass on a dry basis). In general, the composition data were in agreement with the literature [1,44,45]. Mycotoxicological analysis did not detect the presence of aflatoxins or ochratoxin.

The average diameters of the PPC particles used in the extraction tests were 1635 ± 22 µm. Table S1 in the Supplementary material shows the particle size distribution, while Figure S1 shows the appearance of the PPC.

To calculate the bed porosity (Ɛ) of PPC, the apparent density (565 ± 2 kg/m^3^) and true density (792 ± 1 kg/m^3^) were used. Thus, the porosity of the bed of PPC particles in the extractions was 28.7 ± 0.1%.

The TPC determined in the PPC was 5.4 ± 0.1 mg GAE/g PPC, while the FLA content obtained was 2.9 ± 0.1 mg CE/g PPC. There is a scarcity of data in the literature on the contents of minority compounds in the PPC. Attree et al. [46] reported TPC levels in the tegument between 97.29 and 134.36 mg GAE/g sample; for the cotyledon, the authors reported values between 0.88 and 1.85 mg GAE/g sample. Regarding the total flavanol content, the authors reported values between 31.68 and 85.17 mg CE/g sample for the tegument and between 0.18 and 0.30 mg CE/g sample for the cotyledon. The TPC and FLA values determined in this study were intermediate to those reported in the literature for the cotyledon and tegument, probably because the PPC used showed a cotyledon with peanut skin residues.

### 3.2. Single-Stage Batch Extractions of Oil from PPC Using Ethanol as Solvent

As described in Section 2.2.3, preliminary oil extraction experiments were performed from PPC using ethanol as a solvent to evaluate the effects of temperature on the oil extraction yield and NSI of the protein fraction contained in the DSs. The results obtained at 60, 75 and 90 °C in the single-stage extractions with ethanol are presented in Table 1.

The increase in temperature provided a relatively low content of residual lipids in the DSs and, consequently, a relatively high extraction yield. Arnold and Choudhury [20] evaluated the extraction of peanut oil with ethanol at 43 and 72 °C and with hexane at 43 °C. The authors found that complete miscibility of the oil in absolute ethanol was achieved at 72 °C, which corroborated the study by Rao et al. [47], who reported the temperature of 70 °C as sufficient for the complete solubilization of the oil in this solvent. The use of high temperatures in the solid/liquid extraction operation decreased the viscosity of the oil contained in the oleaginous matrix while increasing its solubility and diffusion, providing an increase in the mass transfer coefficient of the washing step [48]. The characteristics of the oleaginous solid material, such as its porosity, solid/solvent ratio and number of contact stages, directly influenced the efficiency of the process.

In Table 1, it is possible to observe an increase in the protein content in the DSs because of the increase in extraction temperature due to oil extraction. In the evaluated temperature range, a maximum NSI value was observed at 75 °C. The parameter indicated the degree of protein denaturation during the process; that is, the lower the NSI value was, the more affected the protein fraction. Protein denaturation could be caused both by the increase in temperature and the use of ethanol [18]. Alcohols could destabilize proteins, weakening hydrophobic interactions, decreasing their solubility, and influencing the subsequent process of obtaining protein concentrates/isolates from DSs.

The protein content and the NSI of the DSs were monitored because this material could be used as a source to obtain protein concentrates or isolates in future studies. Therefore, it was decided to extract the remaining oil from the PPC with alcoholic solvent at 75 °C due to the obtained yield and the high NSI value.

### 3.3. Sequential Oil Extraction from PPC Using Ethanol and Hexane

**Figure 1:**
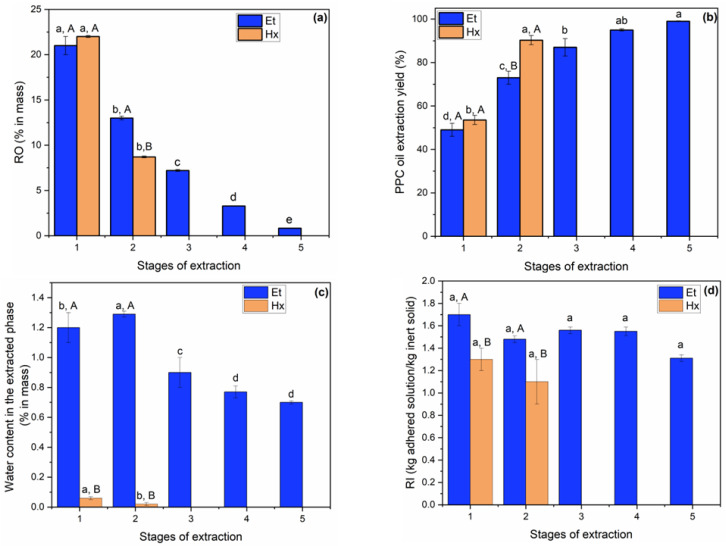
Extraction of oil from PPC with ethanol and hexane as a function of the number of sequential stages: (**a**) residual oil (RO, % by mass) in the DSs; (**b**) cumulative yield of PPC oil extraction (Y, %); (**c**) water content in the extracts (% by mass); and (**d**) retention index (RI, kg adhered solution/kg inert solid). The same lowercase letters for the same solvent show no significant difference by Duncan’s test, and the same uppercase letters for the same extraction stage show no significant difference by the *t*-test (*p* > 0.05).

Figure 1 illustrates the results of extraction of the oil present in the PPC using Et and Hx as solvents.

Figure 1a shows the results of RO in the solid phase as a function of the number of stages evaluated for each solvent: five consecutive stages for Et and two stages for Hx. The RO values decreased as the number of solid/solvent contact stages increased. Consequently, the accumulated oil extraction yield increased as a function of the number of stages evaluated for each solvent (Figure 1b). For Hx, two extraction stages were sufficient to reach an extraction yield of 86 ± 2%, obtaining a defatted solid (DSHx) with 8.7 ± 0.1% residual oil. For Et, to reach a statistically equal level of extraction, three stages were necessary (87 ± 4%). In this case, for the DSEt, the RO value obtained was 7.2 ± 0.1%.

As shown in Figure 1b, it was still possible to observe that the insertion of the fourth and fifth extraction stages with Et did not allow a significant increase in the extraction yield compared to the third stage (*p* > 0.05). Thus, three extraction stages were sufficient for the solvent Et.

The solute–solvent dissolution implied, initially, energy loss in the solute–solute and solvent–solvent interactions and, later, energy gain in the solute–solvent interactions. Thus, the solute solubility was high if the solute–solvent interaction was stronger than the solute–solute interaction, as with n-hexane [49]. By analyzing the solvents Hx and Et in terms of their dielectric constants (measurement of polarity), Et had a dielectric constant (Di) of 18.13 at 75 °C, while Hx had a value of 1.84 at 55 °C; these values have been calculated according to Wohlfarth [50] for the respective temperatures used in the sequential extractions. Thus, Et was more polar than Hx, which is an important parameter in the determination of the solute–solvent interaction. The highly nonpolar characteristic of Hx increased the affinity and solubilization of triacylglycerols by this solvent. Nonpolar solutes had lower solubility in polar solvents, such as Et, which could be compensated with increasing temperature, a greater amount of solvent and a greater number of stages in the process to deplete the material in terms of lipids [18,49].

According to Johnson and Lusas [49], the more polar a solvent is, the greater its ability to extract compounds with similar polarity, such as proteins, carbohydrates, pigments and water. Figure 1c shows the results of water content in the extracts obtained at each extraction stage, demonstrating the greater ability of Et than Hx to dehydrate PPC, as cited by Cheryan et al. [51].

Figure 1d shows the RI values. According to Araújo et al. [52], the RI is directly affected by the affinity of the liquid phase for the solid and inversely proportional to the speed with which the liquid percolates through the solid bed. The parameter is relevant for extractor designs due to its influence on the number of contact stages required to deplete the lipids from the solid matrix. RI influences the amount of heat required for the desolventization of the DS obtained from the extraction process [52].

Throughout the sequential extraction stages, the RI values for Et and for Hx did not show statistically significant differences (*p* > 0.05). In contrast, the RI values for Hx were lower than those for Et (*p* ≤ 0.05), corroborating the data by Araújo et al. [52]. This phenomenon possibly occurred because, due to its polarity, Et interacted more with the surfaces of solids with polar components, such as carbohydrates and proteins [52].

Another factor that influences the RI values is the submission of the oilseed raw material to pretreatments to obtain pellets, collets or flakes. Navarro and Rodrigues [18] evaluated the effects of absolute ethanol on the extraction of oil from the macadamia nut press cake without pretreatment. The authors obtained an RI value at 75 °C of approximately 2.75 kg of adhered solution/kg of inert solids. Rittner [53] compared the effects of different soybean pretreatments (expansion and lamination) on oil extraction. The author obtained an RI of 0.40 kg of adhered solution/kg of inert solids for soybean pellets subjected to oil extraction with Hx and 0.43 kg of adhered solution/kg of inert solids for soybean pellets subjected to oil extraction with Et. The RI values for pretreated materials were lower than those for materials not subjected to pretreatments, such as cakes from pressing, regardless of the solvent used in the solid/liquid extraction step.

**Figure 2:**
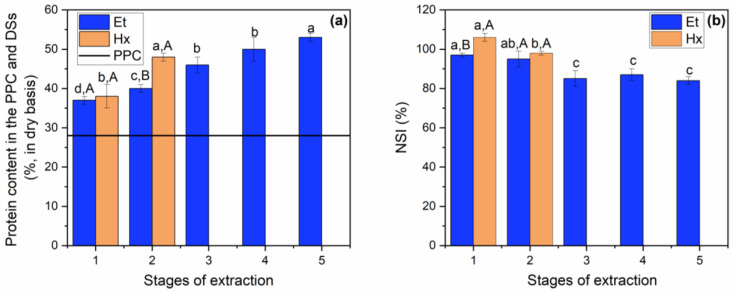
Extraction of PPC oil with ethanol and hexane as a function of the number of sequential stages: (**a**) protein content in the defatted solids (% weight, dry basis); (**b**) nitrogen solubility index (NSI, %) of the defatted solids. The same lowercase letters for the same solvent show no significant difference by Duncan’s test, and the same uppercase letters for the same extraction stage show no significant difference by the *t*-test (*p* > 0.05).

As discussed in Section 3.2, the protein and NSI contents of the DSs obtained at each extraction stage were evaluated to verify the impacts of the sequential stages and solvents on protein solubility. As shown in Figure 2a, while the material was depleted relative to its lipid content (Figure 1a), the protein content proportionally increased. Regarding the NSI of the solids from the extraction with Et, the values decreased from the second stage (*p* ≤ 0.05) and were statistically equal for the third, fourth and fifth extraction stages. For the solids obtained with Hx, there was a decrease (*p* ≤ 0.05) in the NSI in the second stage (Figure 2b), and this value was similar to that obtained with Et for the second stage (*p* > 0.05). In general, the peanut proteins contained in DSEt and DSHx were hardly affected by the solvents, the number of contact stages and the extraction temperature, presenting a high NSI value (greater than 80%). This NSI value was higher than those reported for proteins contained in the DSs of baru kernels with ethanol at 60 °C (NSI of 35% in three stages of oil extraction) [16] and in DSs of macadamia nuts with ethanol at 75 °C (NSI of 35% in four stages of oil extraction) [18].

Based on the above data, in terms of oil extraction yield, it was observed that two stages of extraction for Hx and three stages of extraction for Et were sufficient to achieve statistically equal extraction yield values. To obtain the same extraction yield as Hx, the Et extraction required an additional contact stage, with the use of higher temperature and a greater amount of solvent relative to the solid. These conditions made it possible to obtain DSs with similar protein contents. Thus, to compare the oils extracted with Hx and Et relative to the oil from the mechanical pressing of the peanut grains, in addition to comparing the defatted solids obtained from the extraction with the solvents, further characterizations of the oils and DSs were performed, as presented in Section 3.4 and Section 3.5.

### 3.4. Peanut Oil Characterization

The additional characterization of the peanut cake oils extracted with Et at 75 °C in three sequential stages and Hx at 55 °C in two sequential stages was performed. These oils were named POEt and POHx, respectively, and were compared to peanut oil obtained by cold pressing (CPPO).

The fatty acid profile and the probable TAG compositions are presented in Table 2 and Table 3, respectively, for CPPO, POEt and POHx. The fatty acid compositions of the oils obtained were consistent with the data presented by Davis et al. [4] and Dong et al. [54], which is characteristic of high oleic peanuts. The enrichment in oleic acid content could be achieved through genetic breeding techniques, and the peanut grains were considered high-oleic when the ratio between oleic and linoleic fatty acids (O/Li) had a value ≥ 9 [55]. In the oils obtained, this ratio was 13.0 ± 0.2 for CPPO, 16.3 ± 0.8 for POEt and 16 ± 2 for POHx, with uncertainties estimated by error propagation.

Oleic acid was monounsaturated and less susceptible to peroxidation than polyunsaturated fatty acids. Thus, the high O/Li ratio, with a low IV, could increase the oxidative stability of the high-oleic oil [6].

According to Pattee [5], the IV could vary between 86 and 107 g iodine/100 g of oil for regular peanuts. According to the author, the oil obtained from high-oleic peanuts could have IV values between 73 and 77 g iodine/100 g of oil. The values obtained were higher than those reported for high-oleic peanuts but lower than those reported for regular peanuts (Table 2). In general, the oils obtained by solvent extraction showed similar compositions to those obtained by pressing (*p* > 0.05).

Regarding the probable composition of TAGs, the majority of TAGs estimated in this study were OOO, OOP and OLiO. For Dong et al. [54], the major TAGs were OOO and OOP. The authors reported a reduced percentage of linoleic acid (C18:2), which could contribute to the reduced number of TAGs with this fatty acid in their composition.

According to Akhtar et al. [6], highly mature peanut grains could contain up to 0.5% free acidity (FA), which could be affected by several factors involved in the handling and storage of the raw material. Table 3 shows that the free acidity of CPPO presented a value lower than 0.5%. Conversely, the use of higher temperatures in the solvent extraction step, with the presence of water, could favor the hydrolysis of the TAGs, increasing the free fatty acid content, as observed for the POEt and POHx oils.

In addition to these factors, the use of alcoholic solvents favored the production of oil with high free acidity. Due to the difference in polarity of the solvents, Et had a greater ability to extract polar compounds, including free fatty acids [7]. This solvent could favor alcoholysis, corresponding to the exchange of the acyl group between acylglycerols and ethanol, thus increasing FA [56]. Compared with POHx, the higher amount of solvent and higher number of contact stages could have contributed to the higher FA value in POEt.

The type of extraction affected the luminosity and color of the oils obtained (Table 3). The use of solvents led to differences in the shade of the oils, possibly due to the extraction of chlorophyll and carotenoids, which are common pigments in vegetable oils. Solvent extraction could facilitate the selective leaching of some types of pigments, unlike oil expression by pressing, in which the pigments would be expelled from the solid matrix with the lipids [3]. In addition, Maillard reaction products could contribute to oil browning [57].

The oils obtained from solvent extractions showed increased values for the red/green coordinate (a*) and yellow/blue coordinate (b*); therefore, the oils presented intense coloration (Figure S2 in the Supplementary material). It could be inferred that this color intensification occurred due to the great extraction of pigments. The larger color changes (increase in ΔE) for POEt than for POHx (*p* ≤ 0.05) could have occurred due to the browning reaction caused by the high oil extraction temperature used for Et. The use of Et provided a decrease in luminosity (L*) and an increase in the yellow hue relative to POHx (*p* ≤ 0.05). In general, the oils from solvent extractions should probably be subjected to refining due to their intense color and high FA. Extraction with Et could possibly burden the bleaching and deacidification steps, because the values obtained for color and FA were higher than those for Hx.

Certain data, such as refractive index and density, could be used to quickly differentiate between grains containing high-oleic peanut oil and regular peanut oil. Davis et al. [55] reported refractive index data in oils with O/Li ratios ranging between 12 and 16. The refractive index values reported by the authors ranged between 1.4686 and 1.4688, which are in agreement with the values reported in Table 3.

**Figure 3:**
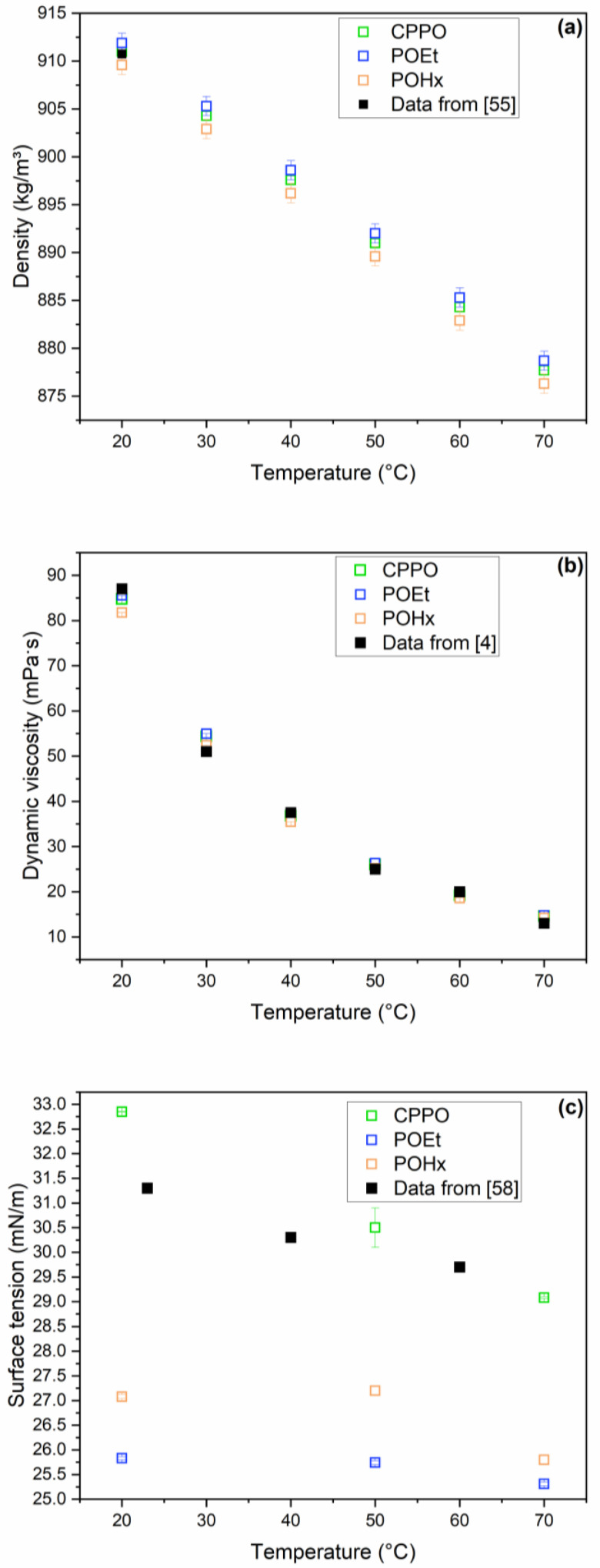
Physical properties of peanut oils obtained by cold pressing (CPPO) and by extraction with ethanol (POEt) and hexane (POHx) solvents as a function of temperature: (**a**) density (kg/m^3^); (**b**) dynamic viscosity (mPa·s); and (**c**) surface tension (mN/m).

Figure 3 presents physical property data for CPPO, POEt and POHx. Figure 3a shows the density values of the oils obtained by solvent extraction and pressing as a function of temperature; the density decreased linearly with increasing temperature (R^2^ ≥ 0.999), regardless of the mode of obtaining the oil. The density values ranged from 911.9 to 876.3 kg/m^3^ in the temperature range from 20 to 70 °C. Davis et al. [55] reported a density of 910.75 kg/m^3^ at 20 °C in peanut oil with an O/Li ratio of 16 (Figure 3a). Comparing the authors’ data with the values obtained, it was observed that, despite the differences between the O/Li ratios, the density values were consistent, regardless of the extraction method.

Figure 3b shows that the dynamic viscosity decreased exponentially with increasing temperature. By comparing the viscosity data determined for CPPO, POEt and POHx with those presented by Davis et al. [4], it was noted that, regardless of the extraction method, the values were consistent.

Figure 3c shows surface tension data for CPPO, POEt and POHx compared to the data reported by Sahasrabudhe et al. [58] determined at 23, 40 and 60 °C for commercial peanut oil. The data for CPPO are consistent with those in the literature, while the surface tension data for the oils obtained by solvent extraction show lower values. It could be inferred that the lower surface tension values determined for POEt and POHx than for CPPO could have appeared due to the lower amount of water present in these oils, which were subjected to drying in a vacuum oven (Table 3). It is possible to observe that the higher the moisture content of the oils, the higher the surface tension value; that is, CPPO > POHx > POEt (Table 3, Figure 3c). Gandova et al. [59] determined surface tension data for water in the temperature range of 20 to 50 °C; the surface tension ranged from 71 to 69 mN/m. The authors observed that the decrease in water content in systems containing ethanolic extracts of sunflower seed bran could contribute to the decrease in the surface tension of the systems, which corroborates the observations of the present study.

### 3.5. Peanut Defatted Solid Characterization

The DSs obtained from sequential three-stage Et extraction at 75 °C (DSEt) and two-stage Hx extraction at 55 °C (DSHx) were characterized by centesimal composition. DSEt and DSHx showed 14.5 ± 0.2 and 9.9 ± 0.6 of lipids, 45.2 ± 0.3 and 47.5 ± 0.4 of proteins, 3.5 ± 0.1 and 3.7 ± 0.2 of ash (% mass in dry basis), 4.9 ± 0.1 and 2.8 ± 0.1% of moisture, and NSI values of 86 ± 6 and 98 ± 1%, respectively. The TPC in DSEt and DSHx was 5.8 ± 0.3 and 9 ± 1 mg GAE/g sample, respectively, while the FLA content corresponded to 1.9 ± 0.1 mg EC/g sample for DSEt and 2.7 ± 0.2 mg EC/g sample for DSHx.

Aiming to assess whether the type of solvent affected the TPC and FLA contents, the results were considered lipid-free, both for the DSs and for the PPC. The TPC for PPC, DSEt, and DSHx were 10.1 ± 0.2, 6.8 ± 0.4, and 9.9 ± 0.8 mg GAE/g defatted sample, respectively, while the FLA contents were 5.4 ± 0.2, 2.3 ± 0.1 and 3.1 ± 0.2 mg CE/g defatted sample, respectively. It is possible to observe lower TPC and FLA values for DSEt compared to DSHx and PPC. It can be inferred that this decrease is because Et can extract phenolic compounds, since these compounds are easily solubilized by solvents with greater polarity [60]. However, the compounds may also have suffered degradation due to ethanol’s higher temperature in the lipid extraction step. According to a study by Liyana-Pathirana and Shahidi [61], using higher temperatures can facilitate the mobility of active compounds. However, it can also promote the degradation of these compounds.

In general, both solvents resulted in solids with reduced oil contents enriched in proteins with high NSI values, enabling their subsequent use for protein extraction.

## 4. Conclusions

The solvents hexane and ethanol were evaluated for the extraction of residual oil from peanut press cake. Ethanol exhibited a similar extraction capacity to hexane; however, the extraction conditions considering the multistage cross-current configuration were different. For hexane, to obtain an extraction yield of 86 ± 2%, two sequential stages were required at 55 °C, with a solid/solvent mass ratio of 1/4. During ethanol extraction, to achieve a yield of 87 ± 4%, it was necessary to use a relatively high temperature (75 °C), a great amount of solvent (solid/solvent 1/5) and three contact stages.

The physical–chemical characterization of the extraction products with solvents, oil and defatted solid allowed us to predict necessary changes in the subsequent operations of oil refining and use for the defatted solid. The solids defatted with ethanol and hexane showed high protein content (>45% by mass) and high nitrogen solubility (>80%), indicating that they could be used to obtain proteins. The oils obtained from extraction with ethanol and hexane presented a composition in terms of fatty acids and triacylglycerols similar to the oil extracted by cold pressing, with triolein as the majority triacylglycerol and a mass ratio between unsaturated and saturated fatty acids greater than 6. The viscosity was similar, ranging from 876 to 912 kg/m^3^ and from 14 to 86 mPa·s, respectively, in the temperature range from 20 to 70 °C. Conversely, the oils obtained from the extractions with the solvents presented lower values of surface tension than the oil obtained from pressing, possibly due to the lower humidity. In general, the oils obtained from solvent extraction showed higher acidity and more pronounced color than the oil extracted by cold pressing. The oil obtained with ethanol presented free acidity values that were 44% and 71% higher than the acidity present in the oils obtained with hexane and by pressing, respectively. Regarding coloration, the green/yellow hue in the oil extracted with ethanol was more intense than in the oils obtained with hexane and by pressing.

Based on these results, it could be inferred that the renewable solvent ethanol could replace hexane in the extraction of residual oil present in the peanut press cake, adding value to this production chain. To enable the correct transition from fossil to renewable solvent use, changes in the solid-liquid extraction and oil refining steps must be considered.

## Figures and Tables

**Table 1:** Oil extraction yield (Y, %), residual oil content (RO, %), protein content (% by mass) and nitrogen solubility index (NSI, %) of the defatted solids obtained in extractions in a single stage with ethanol at different temperatures.

Temperature (°C)	Y (%)	RO (Mass%)	Protein (Mass%)	NSI (%)
60	28 ± 1 ^C^	28 ± 1 ^A^	31 ± 1 ^C^	91 ± 2 ^B^
75	53 ± 3 ^B^	20 ± 1 ^B^	37 ± 1 ^B^	97 ± 1 ^A^
90	69 ± 2 ^A^	14 ± 1 ^C^	43 ± 2 ^A^	83 ± 3 ^C^

The values are means ± standard deviations. The same capital letters in the same column indicate no significant difference by the Duncan test (*p* > 0.05).

**Table 2:** Fatty acid composition (mass %) of peanut oil obtained by cold pressing (CPPO), and by extraction with ethanol (POEt) and hexane (POHx) solvents.

Fatty Acid	Cx:y *	CPPO	POEt	POHx	Davis et al. [4]	Dong et al. [54]
Palmitic (P)	(C16:0)	5.5 ± 0.1 ^C^	6.23 ± 0.01 ^A^	5.9 ± 0.1 ^B^	4.64–5.65	6.19–6.73
Stearic (S)	(C18:0)	2.1 ± 0.1 ^B^	2.68 ± 0.03 ^A^	2.7 ± 0.1 ^A^	1.85–2.31	3.38–6.08
Oleic (O)	(C18:1)	79.5 ± 0.1 ^A^	80.1 ± 0.5 ^A^	79 ± 1 ^A^	79.84–80.12	76.31–80.08
Linoleic (Li)	(C18:2)	6.1 ± 0.1 ^A^	4.9 ± 0.4 ^A^	5 ± 1 ^A^	2.57–3.74	1.47–3.56
Linolenic (Le)	(C18:3)	nd	nd	0.1 ± 0.2	nd	0.42–0.69
Arachidic (A)	(C20:0)	1.01 ± 0.02 ^B^	1.08 ± 0.04 ^AB^	1.17 ± 0.01 ^A^	1.12–1.37	1.51–2.23
Gadoleic (Ga)	(C20:1)	1.81 ± 0.01 ^A^	1.69 ± 0.02 ^B^	1.72 ± 0.01 ^B^	1.93–2.56	0.99–1.44
Behenic (Be)	(C22:0)	2.26 ± 0.01 ^B^	2.08 ± 0.03 ^C^	2.37 ± 0.01 ^A^	2.99–3.34	2.55–3.37
Erucic (E)	(C22:1)	0.1 ± 0.1	nd	nd	nd	nd
Lignoceric (Lg)	(C24:0)	1.53 ± 0.01 ^A^	1.24 ± 0.05 ^B^	1.52 ± 0.02 ^A^	1.93–2.30	1.46–1.65
Average molar mass (g·mol ^−1^*)*		283.7 ± 0.1	283.2 ± 0.1	283.63 ± 0.03	-	-
Iodine value (IV) (g iodine/100 g oil)		82.1	80.2	80.8	-	-
Saturated fatty acids (mass%)		12.4	13.3	13.6	-	15.62–19.62
Unsaturated fatty acids (mass%)		87.6	86.7	86.4	-	80.38–84.38
Unsaturated/Saturated mass ratio		7.0	6.5	6.4	-	4.1–5.4

* Cx:y, x = number of carbons and y = number of double bonds. Nd = not detected. The values are means ± standard deviations. The same capital letters in the same row indicate no significant difference by the Duncan test (*p* > 0.05).

**Table 3:** Probable composition in triacylglycerols (mass %) and quality indices of peanut oils obtained by cold pressing (CPPO), and by extraction with ethanol (POEt) and hexane (POHx) solvents.

	Group ^a^	Triacyl Glycerol ^b^	CPPO	POEt	POHx	Dong et al. [54]
TAG composition	50:1	POP	0.72 ^C^	0.94 ^A^	0.84 ^B^	0.95–1.38
	52:1	POS	0.53 ^C^	0.77^A^	0.74 ^B^	0.37–0.76
	52:2	OOP	12.18 ^C^	14.01 ^A^	13.07 ^B^	9.26–10.30
	52:3	POLi	1.87 ^A^	1.72 ^C^	1.79 ^B^	0.50–1.04
	54:2	OOS	4.86 ^C^	6.17 ^A^	6.11 ^B^	5.97–10.52
	54:3	OOO	51.63 ^B^	52.45 ^A^	50.99 ^C^	59.60–67.81
	54:4	OLiO	11.80 ^A^	9.56 ^C^	10.37 ^B^	0.40–1.00
	54:5	LiLiO	0.91 ^A^	0.59 ^C^	0.90 ^B^	0.03–0.29
	56:1	POBe	0.58 ^C^	0.64 ^B^	0.70 ^A^	0.37–0.69
	56:2	OOA	2.21 ^C^	2.38 ^B^	2.53 ^A^	3.43–4.80
	56:3	OOGa	3.59 ^A^	3.35 ^C^	3.40 ^B^	0.95–1.40
	56:4	OLiGa	0.61	nd	nd	0.03–0.07
	58:1	POLg	0.52 ^C^	0.54 ^B^	0.62 ^A^	0.23–0.26
	58:2	OOBe	4.26 ^B^	3.97 ^C^	4.46 ^A^	4.18–7.17
	58:3	OLiBe	0.89 ^A^	0.54 ^C^	0.66 ^B^	0.24–0.39
	60:2	OOLg	2.85 ^A^	2.35 ^C^	2.83 ^B^	nd
Free acidity (mass%)			0.43 ± 0.02 ^C^	1.47 ± 0.03 ^A^	0.82 ± 0.04 ^B^	
Refractive index (25 °C)			1.47 ± 0.01 ^B^	1.4671 ± 0.0002 ^A^	1.4672 ± 0.0001 ^A^	
Moisture (mass%)			0.32 ± 0.02 ^A^	0.02 ± 0.01 ^C^	0.17 ± 0.01 ^B^	
Color characteristics	L*		91.54 ± 0.04 ^A^	84 ± 3 ^B^	89.4 ± 0.1 ^A^	
	a*		−1.00 ± 0.03 ^A^	−3.3 ± 0.2 ^B^	−3.00 ± 0.01 ^B^	
	b*		3.4 ± 0.2 ^C^	41 ± 1 ^A^	18.6 ± 0.1 ^B^	
	ΔE		nd	39 ± 1 ^A^	15.6 ± 0.1 ^B^	
	°h		−81.49 ± 0.01 ^A^	−85.5 ± 0.5 ^B^	−80.76 ± 0.03 ^A^	

^a^ x:y, x = number of carbons (except for glycerol carbons) and y = number of double bonds. ^b^ Groups with total triacylglycerol composition lower than 0.5% were ignored. Nd = not detected. The values are means ± standard deviations. The same capital letters in the same row indicate no significant difference by the Duncan test (*p* > 0.05).

## Data Availability

The data presented in this study are available on request from the corresponding author.

## Supplementary Materials

The following supporting information can be downloaded at: https://www.mdpi.com/article/10.3390/foods12152886/s1, Table S1: Particle size distribution of peanut press cake. Figure S1: Peanut press cake (PPC). Figure S2: Peanut oils obtained by cold pressing (CPPO), solvent extraction with hexane (POHx), and ethanol (POEt).
