# Supplementary material for: Experimental Comparison between Ethanol and Hexane as Solvents for Oil Extraction from Peanut Press Cake
Source: Foods. 2023 Jul 29;12(15):2886. doi: 10.3390/foods12152886 (PMC10417385; doi:10.3390/foods12152886)
Supplement: Supplementary file 1 [file foods-12-02886-s001.zip › foods-2502861-supplementary.pdf]

Experimental comparison between ethanol and hexane as solvents for oil extraction from peanut press cake

Paloma Jamily Cristina Magalhães [1], Daniel Gonçalves [2], Keila Kazue Aracava [1], and Christianne Elisabete da Costa Rodrigues [1,] *

Table S1. Particle size distribution of peanut press cake (PPC).

d_i (μm)	d_{i+1} (μm)	PPC (g)	Frequency (%)	Average particle size (μm)
2360	3350	2.2 ± 0.3	11 ± 1	1635 ± 22
2000	2360	2.8 ± 0.2	15 ± 1	
1680	2000	3.5 ± 0.1	18.5 ± 0.8	
1190	1680	8.8 ± 0.2	46 ± 1	
841	1190	1.8 ± 0.3	10 ± 2	
595	841	0.020 ± 0.001	0.105 ± 0.002	

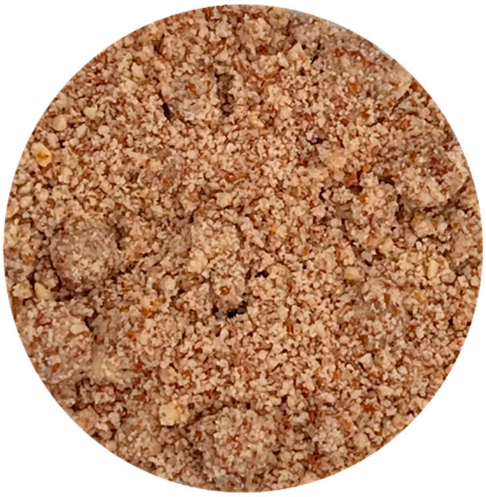

Figure S1. Peanut press cake (PPC).

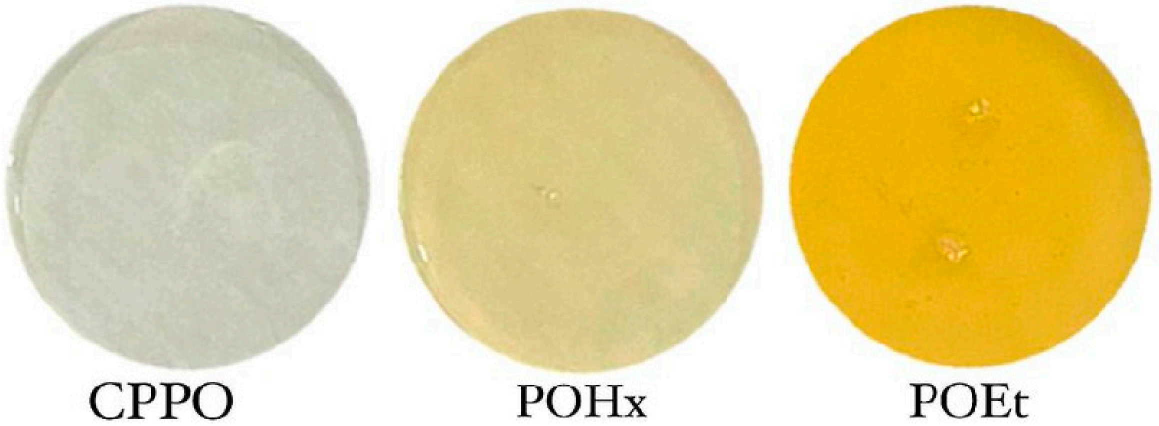

Figure S2. Peanut oils obtained by cold pressing (CPPO), solvent extraction with hexane (POHx), and ethanol (POEt).
